# ﻿Revision of the Neotropical genus *Trigava* O’Brien, 1999 (Hemiptera, Fulgoromorpha, Dictyopharidae, Nersiini), with descriptions of two new species from Peru and Brazil

**DOI:** 10.3897/zookeys.1188.89881

**Published:** 2024-01-03

**Authors:** Zhi-Shun Song, Lois B. O’Brien, Igor Malenovský, Jürgen Deckert, Charles R. Bartlett

**Affiliations:** 1 Institute of Insect Resources and Biodiversity, School of Life Sciences, Chemistry & Chemical Engineering, Jiangsu Second Normal University, Nanjing, 210013 China Jiangsu Second Normal University Nanjing China; 2 Department of Entomology, University of Arizona, Forbes 410, PO Box 210036, Tucson, AZ 85721-0036, USA University of Arizona Tucson United States of America; 3 Department of Botany and Zoology, Faculty of Science, Masaryk University, Kotlářská 2, CZ-611 37, Brno, Czech Republic Masaryk University Brno Czech Republic; 4 Department of Entomology, Moravian Museum, Zelný trh 6, CZ-659 37, Brno, Czech Republic Moravian Museum Brno Czech Republic; 5 Museum für Naturkunde, Leibniz Institute for Research on Evolution and Biodiversity, Invalidenstraße 43, 10115, Berlin, Germany Museum für Naturkunde Berlin Germany; 6 Department of Entomology and Wildlife Ecology, University of Delaware, 250 Townsend Hall, Newark, DE 19716-2160, USA University of Delaware Newark United States of America

**Keywords:** Auchenorrhyncha, Dictyopharinae, Fulgoroidea, identification key, *Igava*, Lappidini, morphology, planthopper, South America, taxonomy

## Abstract

The Neotropical planthopper genus *Trigava* O’Brien, 1999 (Hemiptera, Fulgoromorpha, Dictyopharidae, Nersiini) is revised. Four species are included: *T.brachycephala* (Melichar, 1912) (the type species, from Peru), *T.obrieni* Song, Malenovský & Deckert, **sp. nov.** (from Brazil), *T.peruensis* Song, O’Brien & Bartlett, **sp. nov.** (from Peru), and *T.recurva* (Melichar, 1912) (from Bolivia and Peru). Lectotypes are designated for *Igavabrachycephala* Melichar, 1912 and *Igavarecurva* Melichar, 1912. All species are described, including habitus photographs and detailed illustrations of the male genitalia. Male and female genitalia are described for this genus for the first time. A key for identification of the species of *Trigava* and a distribution map are provided.

## ﻿Introduction

The genus *Trigava* O’Brien, 1999 was originally established as a segregate out of the genus *Igava* Melichar, 1912. Melichar (1912) erected *Igava* based on *Dictyopharacallipepla* Gerstaecker, 1895 from Peru (the type species) and described two additional species, *Igavabrachycephala* Melichar, 1912 (from Peru) and *I.recurva* Melichar, 1912 (from Peru and Bolivia). However, O’Brien (1999) disagreed with this arrangement and established a new genus *Trigava* O’Brien, 1999 for the latter two species based on Melichar’s descriptions and illustrations for *I.brachycephala*. She suggested that *Trigava* may be distinguished from *Igava* by the green dorsal marginal carina of the pronotum (not continued on the tegula, absent in *Igava*), the frons of equal width basally and apically, and the shape of the head. Emeljanov (2011) placed *Trigava* in the tribe Nersiini Emeljanov, 1983 and *Igava* in the tribe Lappidini Emeljanov, 1983, respectively. This classification was supported by a morphological phylogenetic analysis by Song et al. (2018).

Based on examination of types and additional specimens, *Trigava* is here revised. We redescribe the genus and Melichar’s (1912) species and add two new species, *T.obrieni* Song, Malenovský & Deckert, sp. nov. (from Brazil) and *T.peruensis* Song, O’Brien & Bartlett, sp. nov. (from Peru). We provide an identification key and photographic illustrations for each species, showing also the structures of the male and female genitalia for the first time, described and illustrated in detail.

## ﻿Material and methods

The specimens studied in the course of this work are deposited in the following institutions, which are subsequently referred to their acronyms: **LBOB**, personal collection of Lois B. O’Brien (now deposited at the University of Arizona, Tucson, Arizona, USA); **MFNB**, Museum für Naturkunde, Berlin, Germany; **MMBC**, Moravské zemské muzeum (Moravian Museum), Brno, Czech Republic; and **MTD**, Museum für Tierkunde, Dresden, Germany.

The post-abdominal segments of the specimens used for dissections were cleared in 10% KOH at room temperature for c. 6–12 hours, rinsed and examined in distilled H_2_O and then transferred to 10% glycerol and enclosed in microvials pinned with the specimens. Observations were conducted under a stereomicroscope, measurements and photography under Leica M205 C stereomicroscopes equipped with a Canon EOS 7D digital camera or a Keyence VHX-5000 digital microscope with VH-Z20T and VH-ZST objectives. Some final images were compiled from multiple photographs using CombineZM 1.0.0 image stacking software and improved with the Adobe Photoshop CS5 software.

The morphological terminology and measurements used in this study follow Song et al. (2016, 2018) for most characters, Bourgoin (1993) for the female genitalia, and Bourgoin et al. (2015) for the tegmen. Species characteristics shared with the generic description are not repeated except for clarity.

## ﻿Taxonomy

### ﻿Family Dictyopharidae Spinola, 1839

**Tribe Nersiini Emeljanov, 1983**

#### ﻿Genus Trigava

Taxon classificationAnimaliaHemipteraDictyopharidae

O’Brien, 1999

79AB2FB5-9227-5448-99F6-8903C2248812

Trigava O’Brien, 1999: 60. Type species: Igavabrachycephala Melichar, 1912; by original designation.

##### Diagnosis.

The genus may be distinguished by the following combination of characters: cephalic process conical, strongly curved upward, and gradually narrowing apicad; vertex with posterior plane elevated above pronotum, wider (e.g., at posterior margin) than transverse diameter of eyes in dorsal view, lateral carinae abruptly constricted and curved upward in front of eyes, converging anteriad, apical margin broadly angulately convex to nearly straight; frons flat, lateral, intermediate and median carinae weakly ridged, lateral carinae nearly parallel in most of their length, gradually converging apicad in front of eyes; pronotum with intermediate carinae ridged and nearly reaching posterior margin, upper lateral carina greenly thickened (not continued on the tegula), posterior margin angularly concave, not notched; mesonotum with lateral carinae incurved anteriad, reaching and connecting median carina; tegulae lacking carina; tegmina macropterous, veins setose on ventral surface, nodal line present, ScP+R+MP long, MP_1+2_, MP_3+4_ and CuA_1_ forked near nodal line (near midlength), the longest folding line between MP_3_ and MP_4_; fore femora without spines, hind tibiae with eight apical teeth; endosomal processes sclerotised apically; phallobase with pairs of large and stout spines.

##### Description.

**Figure 1.**
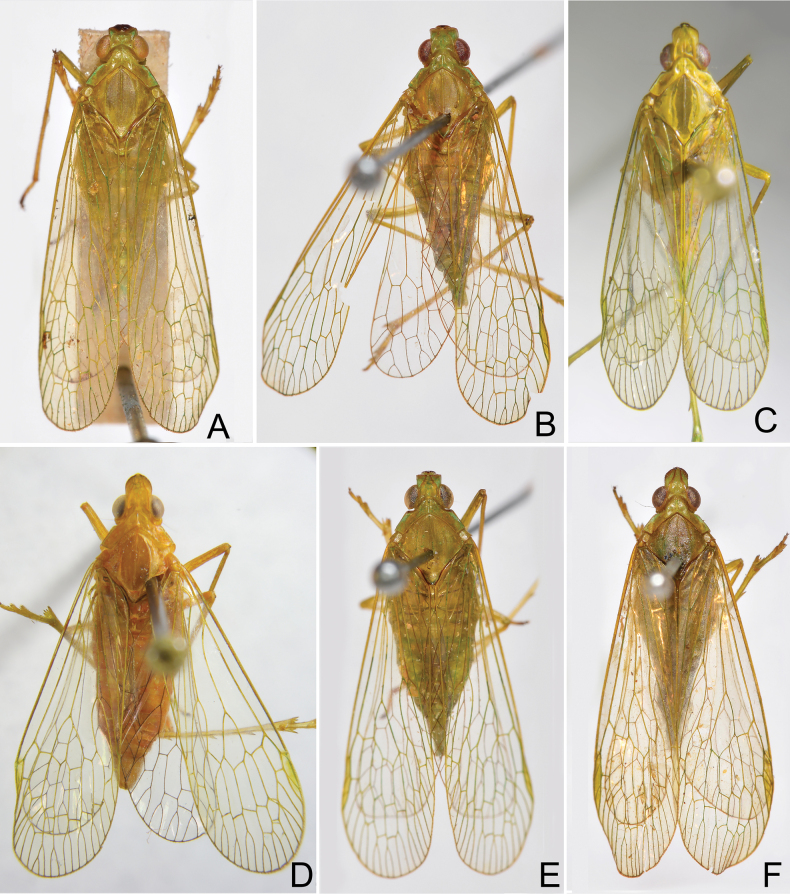
Habitus of *Trigava* species, dorsal view **A***T.brachycephala* (Melichar), lectotype, male **B***T.brachycephala* (Melichar), male **C***T.obrieni* sp. nov., holotype, male **D***T.obrieni* sp. nov., paratype, female **E***T.peruensis* sp. nov., holotype, male **F***T.recurva* (Melichar), lectotype, male.

**Figure 2.**
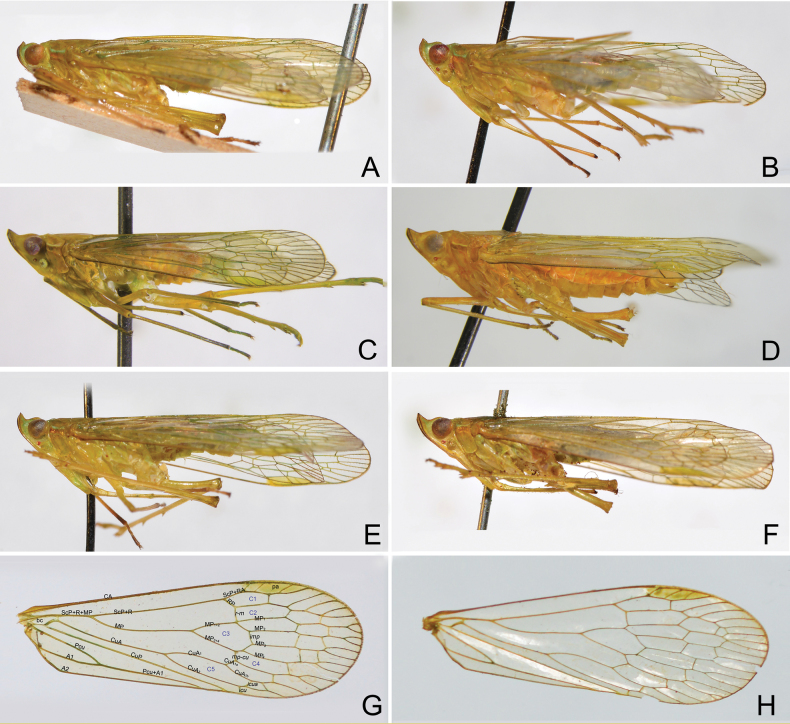
Habitus of *Trigava* species, lateral view **A***T.brachycephala* (Melichar), lectotype, male **B***T.brachycephala* (Melichar), male **C***T.obrieni* sp. nov., holotype, male **D***T.obrieni* sp. nov., paratype, female **E***T.peruensis* sp. nov., holotype, male **F***T.recurva* (Melichar), lectotype, male **G** right tegmen of *T.brachycephala***H** right tegmen of *T.recurva*.

General colour of body pale green to stramineous green, marked with green, ochraceous and black on head and thorax (Figs 1, 2).

**Figure 3.**
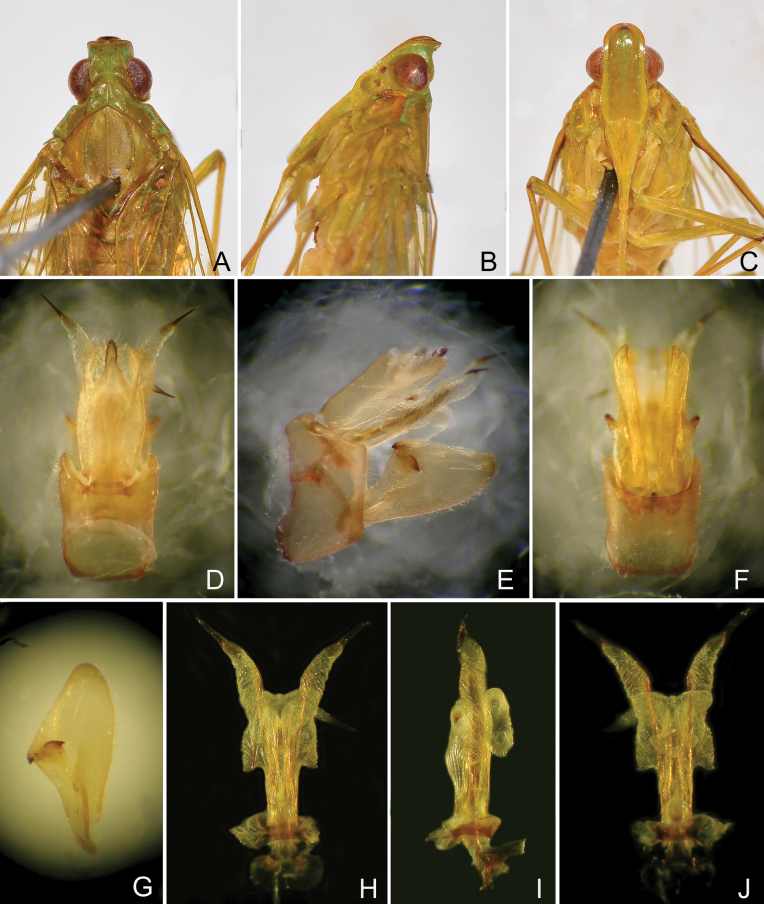
*Trigavabrachycephala* (Melichar), male **A** head and thorax, dorsal view **B** same, lateral view **C** same, ventral view **D** pygofer and segment X, dorsal view **E** pygofer, gonostyles, aedeagus and segment X, right lateral view **F** pygofer and gonostyles, ventral view **G** left gonostyle, lateral view **H** aedeagus, dorsal view **I** aedeagus, lateral view **J** aedeagus, ventral view.

**Figure 4.**
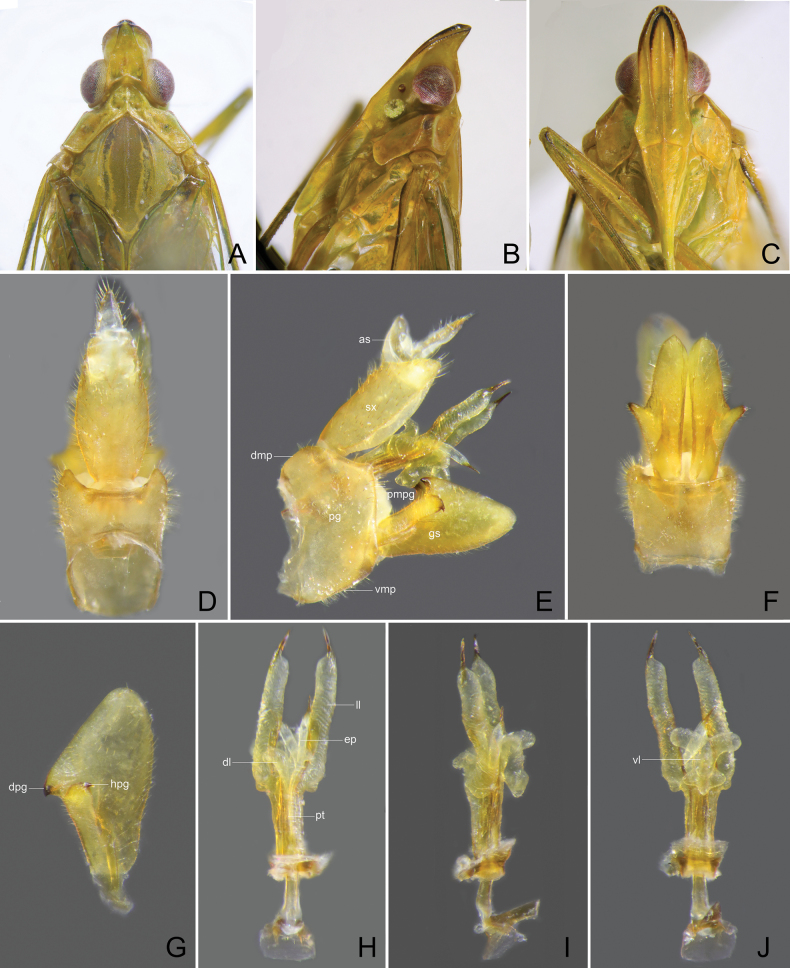
*Trigavaobrieni* sp. nov., holotype, male **A** head and thorax, dorsal view **B** same, lateral view **C** same, ventral view **D** pygofer and segment X, dorsal view **E** pygofer, gonostyles, aedeagus and segment X, right lateral view **F** pygofer and gonostyles, ventral view **G** left gonostyle, lateral view **H** aedeagus, dorsal view **I** aedeagus, lateral view **J** aedeagus, ventral view. Abbreviations: as, anal style; dl, dorsal lobes of phallotheca; dmp, dorsal margin of pygofer; dpg, dorsal process of gonostyle; ep, endosomal processes; gs, gonostyle; hpg, hook-like process of gonostyle; ll, lateral lobes of phallotheca; pg, pygofer; pt, phallotheca; sx, segment X; vl, ventral lobes of phallotheca; vmp, ventral margin of pygofer.

***Head*.** Head (Figs 3A–C, 4A–C, 6A–C, 7A–C) in front of eyes produced into a short cephalic process. Cephalic process conical, strongly curved upward, and gradually narrowing toward apex. Vertex (Figs 3A, 4A, 6A, 7A) with posterior plane elevated above pronotum, wider than transverse diameter of eyes in dorsal view (Figs 3A, 4A, 6A, 7A); lateral carinae keeled, gradually converging (with a lateral inflection anterior to eyes), abruptly constricted and curved upward in front of eyes, and then converging anteriad, in lateral view (Figs 3B, 4B, 6B, 7B), the process bent upward at approximately 60–90° (sometimes more than 90°) in front of eyes; median carina indistinct, somewhat depressed medially, or weakly ridged posteriorly; apical margin broadly angularly convex to nearly straight, not acuminate at apex, posterior margin ridged and broadly and angularly concave, concavity projecting distinctly beyond middle of eyes. Frons (Figs 3C, 4C, 6C, 7C) flat, elongate and relatively broad; lateral carinae weakly ridged, slightly expanded outward below antennae, nearly parallel in most of their length, gradually converging apicad in front of eyes; intermediate carinae weekly keeled, nearly reaching frontoclypeal suture; median carina complete but obscure. Frontoclypeal suture arched. Postclypeus and anteclypeus (Figs 3C, 4C, 6C, 7C) cuneate, slightly convex medially; lateral and median carinae keeled. Rostrum long, second segment slightly longer than third segment, surpassing middle coxae, third segment reaching middle of hind femora. Compound eyes large and rounded, callus postocularis forming a triangular process protruded posteriad. Antenna with very small scape; pedicel subglobose, with more than 50 sensory plaque organs distributed over entire surface; flagellum long, setuliform.

***Thorax*.** Pronotum (Figs 3A, 4A, 6A, 7A) distinctly shorter than mesonotum medially, narrow anteriorly, broad posteriorly; anterior margin arcuately convex medially, lateral marginal areas convex and sloping with two long longitudinal carinae on each side between eyes and tegulae, upper lateral carina greenly thickened (not continued on the tegula); in dorsal view (Figs 3A, 4A, 6A, 7A), lower lateral carinae expanded just behind eyes; posterior margin angularly concave, not notched; median carina distinct, with a deep lateral pit at side; intermediate carinae keeled and nearly complete. Mesonotum (Figs 3A, 4A, 6A, 7A) distinctly tricarinate, lateral carinae incurved anteriad, reaching median carina. Tegulae lacking carina.

Tegmina (Figs 2G, H) membranous, hyaline, and macropterous, extending far beyond the tip of abdomen; veins setose on ventral surface; transverse veinlets *r-m*, *imp* and *mp-cu* connecting some short segments of veins RP, MP and CuA to form nodal line; ScP+R+MP long, much longer than basal cell; ScP+R forked in distal 3/5; MP bifurcating MP_1+2_ and MP_3+4_ (forming cell C3) near midlength, MP_1+2_ and MP_3+4_ forked near level of bifurcation of ScP+R, near nodal line, MP_1_, MP_2_, MP_3_ and MP_4_ each forked at apex; CuA forked before bifurcation of MP (forming C5 cell), CuA_1_ bifurcating CuA_1a_ and CuA_1b_ before *mp-cu*, near nodal line; *mp-cu* connecting MP_3+4_ and CuA_1a_ (forming C4 cell); Pcu and A1 fused in proximal third of clavus, composite vein reaching wing margin before claval apex (not reaching CuP); numbers of apical cells of RP, MP and CuA 3, 7, 3–4, respectively; pterostigmal area elongate, with three cells; folding lines present, the longest one between MP_3_ and MP_4_.

Hindwings with ScP+R+MP short, about a half of basal cell; ScP+RA and RP forked near apical one third; MP bifurcating MP_1+2_ and MP_3+4_ little posterior to ScP+RA and CuA; CuA bifurcating CuA_1_ and CuA_2_ much anterior to ScP+RA and MP, CuA_1_ four-branched distally, and CuA_2_ not branched; transverse veinlets *r–m* and *mp-cu* slightly posterior to bifurcation of MP.

Legs long; fore femora elongate, not flattened and dilated, without spines; fore and middle tarsomeres I and II with more than two acutellae; hind tibiae distinctly elongate, nearly twice as long as hind femora, with four lateral spines and eight apical teeth; hind tarsomeres I and II with about 7–9 and 8–9 apical teeth, respectively.

***Male genitalia*.** Pygofer, in lateral view (Figs 3E, 4E, 6E, 7E), irregularly quadrate to pentagonal, distinctly wider ventrally than dorsally, dorsal margin slightly excavated to accommodate segment X, dorsoposterior margins angular. Gonostyles (Figs 3F, 4F, 6F, 7F) symmetrical, in lateral view (Figs 3G, 4G, 6G, 7G) broad, base narrow, expanded distally, broadest in middle, and then tapering posteriad, apex rounded; dorsal margin with a claw-like, apically sclerotised process (dorsal process), directed dorsad, outer dorsal edge with a hook-like process near middle, directed ventrad. Aedeagus (Figs 3H–J, 4H–J, 6H–J, 7H–J) with a pair of endosomal processes extended from phallotheca and curved dorsoanteriad or laterad; these processes mostly membranous, sclerotised apically, tapering posteriad to form large and stout spines; phallobase sclerotised basally and membranous and inflated apically, with paired lobes with large and stout spines. Segment X long oval, in lateral view (Figs 3E, 4E, 6E, 7E), ventral margin gradually widening from base to apex; in dorsal view (Figs 3D, 4D, 6D, 7D), with apex excavated to accommodate anal style; anal style elongate and large, beyond apical ventral margin of segment X.

**Figure 5.**
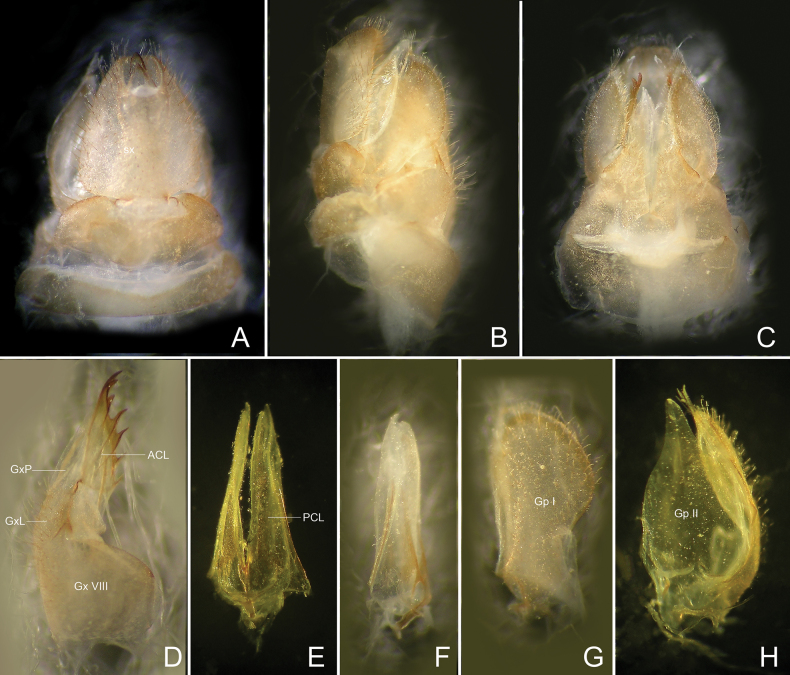
*Trigavaobrieni* sp. nov., paratype, female **A** terminalia, dorsal view **B** same, lateral view **C** same, ventral view **D** gonapophysis VIII, lateral view **E** gonapophysis IX, ventral view **F** gonapophysis IX, lateral view **G** gonoplac, lateral view **H** gonoplac, dorsal view. Abbreviations: ACL, anterior connective lamina of gonapophysis VIII; Gp I, first lobe (lateral lobe) of gonoplac; Gp II, second lobe (posterior lobe) of gonoplac; GxL, endogonocoxal lobe; GxP, endogonocoxal process; Gx VIII, gonocoxae VIII; PCL, posterior connective lamina; sx, segment X.

***Female genitalia*.** Gonocoxae VIII (Fig. 5D) with a membranous and flattened endogonocoxal processes (Gxp), bearing tiny setae on apex. Gonapophyses VIII (Fig. 5D) with anterior connective lamina large and sclerotized, with seven teeth of varying sizes and shapes. Gonapophyses IX (Fig. 5E, F) with posterior connective lamina triangular, symmetrical, weakly bifurcated at apex, fused with the intergonocoxal plate at base. Gonoplacs (Fig. 5G, H) with two lobes homologous, first lobe (lateral lobe) axe-shaped, sclerotized, large and elongate, apical margin filmy and truncate, with cluster of long setae on apex (no sensory appendage, viz. *Igava* spp.); second lobe (posterior lobe) large, broad basally and tapering posteriad, the edge membranous containing long sclerotized plate. Segment X (Fig. 5A) trapeziform, large and broad in dorsal view, apex deeply excavated to accommodate anal style; anal style small.

**Figure 6.**
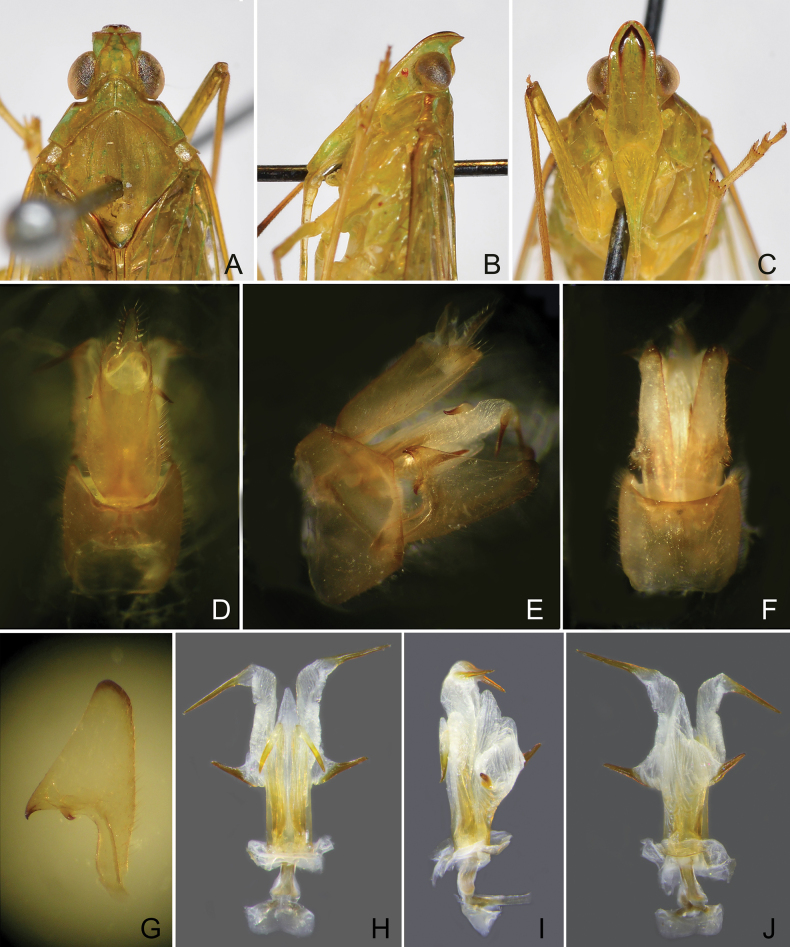
*Trigavaperuensis* sp. nov., holotype, male **A** head and thorax, dorsal view **B** same, lateral view **C** same, ventral view **D** pygofer and segment X, dorsal view **E** pygofer, gonostyles, aedeagus and segment X, right lateral view **F** pygofer and gonostyles, ventral view **G** left gonostyle, lateral view **H** aedeagus, dorsal view **I** aedeagus, lateral view **J** aedeagus, ventral view.

**Figure 7.**
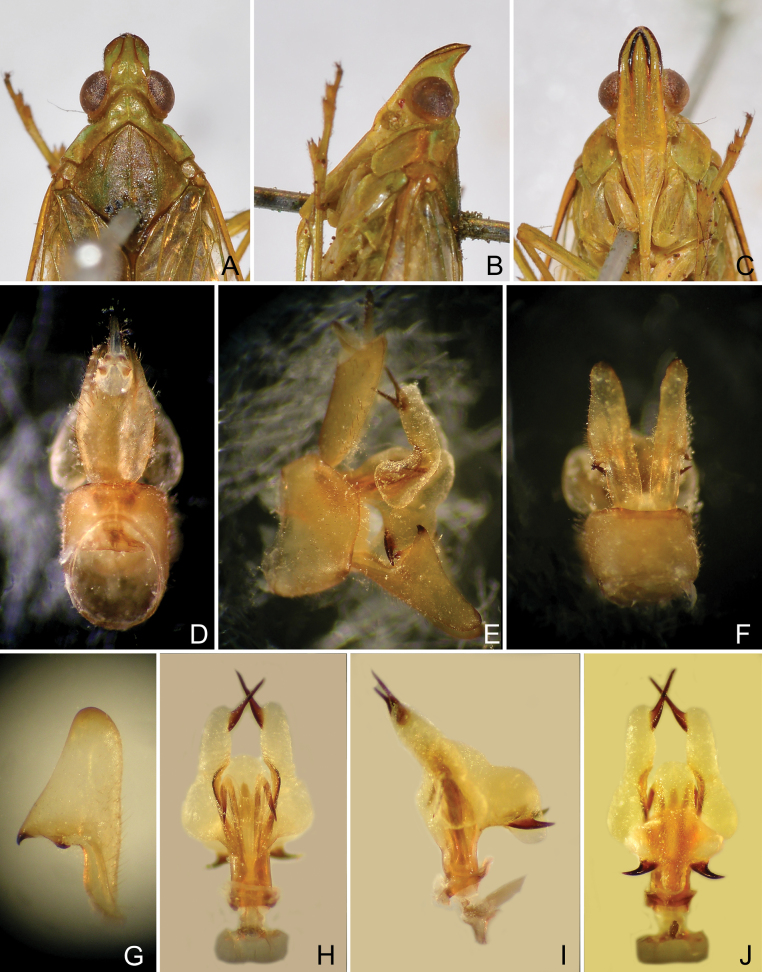
*Trigavarecurva* (Melichar), lectotype, male **A** head and thorax, dorsal view **B** same, lateral view **C** same, ventral view **D** pygofer, and segment X, dorsal view **E** pygofer, gonostyles, aedeagus and segment X, right lateral view **F** pygofer and gonostyles, ventral view **G** left gonostyle, lateral view **H** aedeagus, dorsal view **I** aedeagus, lateral view **J** aedeagus, ventral view.

##### Diversity and distribution.

**Figure 8.**
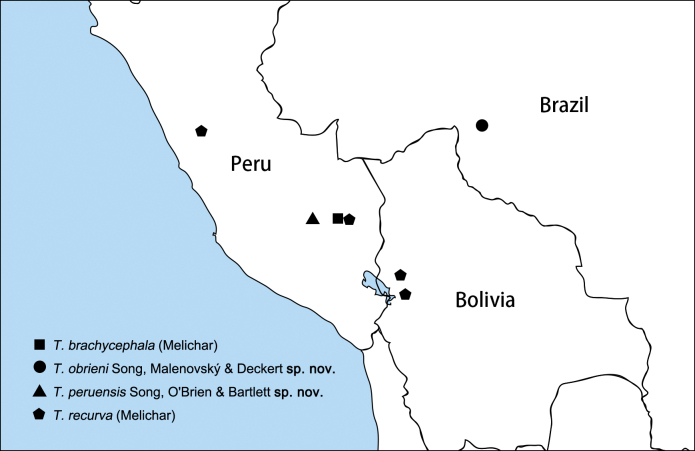
Geographical distribution of *Trigava* species.

*Trigava* is revised here to contain four species including two new species. The species of the genus are distributed in the northwest of South America and were recorded from Peru, Bolivia and Brazil, as far as known (Fig. 8).

##### Remarks.

In addition to the diagnostic characters listed by O’Brien (1999), *Trigava* may be separated from *Igava* Melichar by characters diagnostic at the tribal level, such as on the tegmen and the female genitalia (Song et al. 2018). For example, the second postnodal line of the tegmen and the apical sensory appendage of gonoplacs I are absent in *Trigava*, but they are present in *Igava*.

In the tribe Nersiini, *Trigava* is externally similar to the genus *Nersia* Stål, 1862, but can be distinguished from the latter by the head strongly curved upward (slightly curved upward in *Nersia*); the median carina of vertex absent (present in *Nersia*), and the tegulae lacking carina (present in *Nersia*). See also the Discussion.

### ﻿Key to the species of *Trigava*

**Table:** 

1	Gonostyles with dorsal process short, hook-like process situated submedially, curved basad (Figs 3G, 4G); ventral lobes of aedeagus without spines (Figs 3J, 4J)	**2**
–	Gonostyles with dorsal process elongate, hook-like process situated more basally and curved apicad (Figs 6G, 7G); ventral lobes of aedeagus with long spines (Figs 6J, 7J)	**3**
2	Head with cephalic process very short, extremely curved upward 90° (even more than 90°) in front of eyes (Fig. 3B); ventral lobes of aedeagus with a minute tooth at base (Fig. 3J)	***T.brachycephala* (Melichar)**
–	Head with cephalic process relatively longer, curved upward about 60° in front of eyes (Fig. 4B); ventral lobes of aedeagus without tooth at base (Fig. 4J)	***T.obrieni* Song, Malenovský & Deckert, sp. nov.**
3	Head with cephalic process short, extremely curved upward about 90° in front of eyes (Fig. 6B); ventral lobes of aedeagus weakly trilobed, apex produced in a long triangular lobe (Fig. 6J)	***T.peruensis* Song, O’Brien & Bartlett, sp. nov.**
–	Head with cephalic process relatively longer, curved upward about 60° in front of eyes (Fig. 7B); ventral lobes of aedeagus nearly cross-shaped, base protruded anteriad, and apex produced in a large thumb-like lobe (Fig. 7J)	***T.recurva* (Melichar, 1912)**

#### ﻿ Trigava brachycephala

Taxon classificationAnimaliaHemipteraDictyopharidae

(Melichar, 1912)

37706BA3-005E-5218-982D-F4C4A95E2F27

Figs 1A, B , 2A, B, G , 3A–J

Igava brachycephala Melichar, 1912: 49, pl. II, figs 9, 11.
Igava brachycephala Melichar: Metcalf (1946): 39.
Trigava brachycephala (Melichar): O’Brien (1999): 60.

##### Type locality.

Peru, Department of Cuzco, Quispicanchi Province, Marcapata.

##### Emended description.

***Measurements*** (in mm; 3♂, 1♀). Body length from apex of head to tip of tegmina: ♂ 10.8–11.1, ♀ 11.7; head length (includes: apex of cephalic process to constricted and curved part + from curved part to base of eyes): ♂♀ (0.2–0.3)+(0.6–0.7); head width including eyes: ♂ 1.4–1.5, ♀ 1.6; tegmen length: ♂ 9.0–9.3, ♀ 9.6.

***Coloration*.** Head stramineous green, apical spot between intermediate carinae of frons black, intermediate carinae of frons reddish brown, lateral areas of head green. Pronotum and mesonotum stramineous green, upper lateral carinae of pronotum green. Tegmina and hindwings membrane hyaline, veins green to greenish yellow, pterostigmal area more or less greenish ochraceous. Legs yellowish brown, base, apex and apical spines of tibiae fuscous. Abdomen dorsally and ventrally greenish ochraceous.

***Structure*.** Head with cephalic process very short, in lateral view (Fig. 3B), strongly curved upward about 90° (or more than 90° in lectotype, Fig. 2A) in front of eyes. Vertex (Fig. 3A) broad, with ratio of length at midline to width between eyes (0.9–1.0):1. Frons (Fig. 3C) flat, relatively broad, with ratio of length at midline to maximum width 2.0:1, intermediate and median carinae obscure.

***Male genitalia*.** Pygofer in lateral view (Fig. 3E) with ratio of ventral to dorsal width about 2.5:1; posterior margin produced angulately near middle. Gonostyles (Fig. 3F) large and broad, dorsal process short, acute apically, more or less incurved and directed dorsoanteriad; hook-like process situated submedially, distinctly elevated above dorsal process, curved basad (Fig. 3G). Aedeagus (Fig. 3H–J) elongate, endosomal processes curved dorsoanteriad; phallobase sclerotized and pigmented at lateral sides, membranous and slightly inflated dorsally and ventrally: dorsal lobes V-shaped at apex, directed posteriad; a pair of lateral lobes large and elongate, base conical, directed anterolaterad, mostly tapering apicad, apex with a large long spine, directed posteriad; ventral lobes small, butterfly-shaped, base expanded laterad, with a minute tooth, apex produced in a pair of short and stout lobes, without spine, directed laterad. Segment X (Fig. 3D, E), in dorsal view, lateral margins more or less convex near middle, with ratio of length to width near middle about 2.0:1.

***Female genitalia*.** As in generic description.

##### Type material examined.

***Lectotype*** (here designated), ♂, (1) “Peru, Marcapata”; (2) “*Enh.brachycephala* [Melichar’s handwriting], det. Melichar”; (3) “Typus” [dark red label]; (4) “Collectio Dr. L. Melichar, Moravské museum Brno”; (5) “Syntypus, *Igavabrachycephala* sp.n. Melichar, 1912, ♂ [P. Lauterer’s handwriting], P. Lauterer det. 1991”; (7) “Syn- typus” [red label]; (7) “Invent. č. 4941/Ent., Mor. muzeum, Brno”; (8) “*Trigavabrachycephala* [Zhi-Shun Song’s handwriting] det. Z.S. Song 2014”; (9) “Lectotypus ♂, *Igavabrachycephala* Melichar, 1912, designated by Z.S. Song & I. Malenovský, 2023” [newly added red label] (MMBC; dry-mounted, glued on a rectangular card label, abdomen detached, macerated, preserved in glycerol and enclosed in a glass microvial placed on the same pin as the specimen). ***Paralectotype***, 1♂, (1) “Peru S, Marcapata, Garlepp c.”; (2) “Coll. A. Jacobi, 1912 – 3” [green label]; (3) “*brachycephala*” [handwriting]; (4) “*Igava Mel.*” [handwriting]; (5) “Paralectotypus ♂, *Igavabrachycephala* Melichar, 1912, labelled by I. Malenovský, 2023” [newly added red label] (MTD).

##### Other material examined.

**Peru**: 1♂, “Peru, S.V. Garlepp”; “*Igavabrachycephala* Mel. [H. Synave’s handwriting], H. Synave det., 1969” (MFNB); 1♂, 1♀, “Peru” (MTD).

##### Distribution.

Southeastern Peru.

##### Remarks.

Melichar (1912) described *Igavabrachycephala* Melichar based on material from “Peru, Marcapata (Garlepp) (Mus. Budapest, Dresden)”. He did not state the number of the specimens he used for the description nor did he designate a holotype. One of his syntypes has been preserved in Melichar’s personal collection in MMBC and we used this male specimen for the redescription of the species. According to Article 74 of ICZN (1999), we designate the specimen in MMBC as the lectotype for *I.brachycephala* to stabilize the nomenclature. Another conspecific male with collecting data fully matching the original description has been located by us in MTD and was labelled by us as a paralectotype.

#### ﻿ Trigava obrieni

Taxon classificationAnimaliaHemipteraDictyopharidae

Song, Malenovský & Deckert sp. nov.

474620BB-79E7-5B9B-B9F5-3153619B3DD7

https://zoobank.org/87633FF5-777E-4A66-9F5E-536B76E82C08

Figs 1C, D , 2C, D , 4A–J , 5A–H

##### Type locality.

Brazil, Rondônia State, 62 km SW Ariquemes, Fazenda, Rancho Grande.

##### Type material.

***Holotype*** ♂, **Brazil**: Rondônia, 62 km, SW Ariquemes, Fzda, Rancho Grande, 19-XI-1994, C.W. O’Brien & L.B. O’Brien leg. (LBOB; dry-mounted, pinned). ***Paratypes*: Brazil**: 1♂, 1♀, same data as holotype but 18-XI-1994 (LBOB); 1♂, same data as holotype but 4–16-XI-1997, J.E. Eger leg. (LBOB).

##### Diagnosis.

*Trigavaobrieni* sp. nov. is similar to *T.brachycephala* in most characters, but can be separated from the latter by the longer head curved upward about 60° in front of eyes (in *T.brachycephala*, the cephalic process is distinctly shorter and curved upward more than 90° in front of eyes) and the ventral lobes of the aedeagus without a tooth at the base (with a minute tooth at base in *T.brachycephala*). This new species also may be differentiated from *T.recurva* (Melichar) by the gonostyles with the dorsal process short and the hook-like process situated submedially and curved basad (dorsal process distinctly elongate, hook-like process situated more basally and curved apicad in *T.recurva*), and the ventral lobes of the aedeagus without long spines (with long spines in *T.recurva*).

##### Description.

***Measurements*** (in mm; 3♂, 1♀). Body length from apex of head to tip of tegmina: ♂ 10.8–11.2, ♀ 12.5; head length (includes: apex of cephalic process to constricted and curved part + from curved part to base of eyes): ♂♀ (0.4–0.5)+(1.0–1.1); head width including eyes: ♂ 1.4–1.5, ♀ 1.6; tegmen length: ♂ 8.5–8.9, ♀ 9.8.

***Coloration*.** Head stramineous green, lateral and intermediate carinae of frons in front of eyes black to blackish brown, lateral areas in front of eyes green. Pronotum and mesonotum stramineous green, upper lateral carinae of pronotum green. Tegmina and hindwings with membrane hyaline, costal margin black to dark brown, veins green to greenish yellow, pterostigmal area more or less greenish ochraceous. Legs yellowish brown, base, apex and apical spines of tibiae fuscous. Abdomen dorsally and ventrally greenish ochraceous.

***Structure*.** Head with cephalic process relatively long, in lateral view (Fig. 4B), curved upward about 60° in front of eyes. Vertex (Fig. 4A) broad, with ratio of length at midline to width between eyes (1.5–1.6):1. Frons (Fig. 4C) flat, relatively broad, with ratio of length at midline to maximum width 2.4:1.

***Male genitalia*.** Pygofer in lateral view (Fig. 4E) with ratio of ventral to dorsal width about 1.7:1; posterior margin produced into a small, apically obtuse process near middle. Gonostyles (Fig. 4F) large and broad, dorsal process short, acute apically, more or less incurved and directed dorsoanteriad; hook-like process placed submedially, horizontal with dorsal process, curved basad (Fig. 4G). Aedeagus (Fig. 4H–J) slender and elongate, endosomal processes curved dorsoanteriad; phallobase sclerotized and pigmented at lateral sides, membranous and slightly inflated dorsally and ventrally: dorsal lobes V-shaped at apex, curved and directed posterolaterad; a pair of lateral lobes large and elongate, thumb-like, apex with a long spine, directed posteriad; ventral lobe small, butterfly-shaped, base expanded laterad, apex produced in a pair of thumb-like moderate lobes, without spine, directed laterad. Segment X (Fig. 4D, E), in dorsal view, with lateral margins more or less convex near middle, with ratio of length to width near middle about 2.0:1.

***Female genitalia*.** As in generic description (Fig. 5A–H).

##### Etymology.

The new species is named after the late Dr Charlie W. O’Brien, former professor at Florida Agricultural and Mechanical University, USA, one of the world’s top experts in weevils, collector of the type specimens and husband of Dr Lois B. O’Brien, in recognition of their kindest help and support to the first author when he visited USA in 2017. The species name is to be treated as a noun in genitive case.

##### Distribution.

Northwestern Brazil.

#### ﻿ Trigava peruensis

Taxon classificationAnimaliaHemipteraDictyopharidae

Song, O’Brien & Bartlett sp. nov.

B142DB11-01D8-5A34-93D0-0980E50C5FD6

https://zoobank.org/F2AA6095-E279-4772-A1B8-CE3F0B2774F3

Figs 1E , 2E , 6A–J

##### Type locality.

Peru, Department of Cuzco, Cosñipata Valley.

##### Type material.

***Holotype*** ♂, **Peru**: “Peru, Dep Cuzco, Cosnipata-Ebene, 1000 m, XI-XII-[19]00, S.V. Garlepp leg.” (MFNB; dry-mounted, pinned).

##### Diagnosis.

*Trigavaperuensis* sp. nov. is externally similar to *T.brachycephala*, but can be separated from the latter by the gonostyles with the dorsal process elongate and the hook-like process placed more basally and curved apicad (dorsal process short and hook-like process placed submedially and curved basad in *T.brachycephala*), and the ventral lobes of the aedeagus with long spines (without long spines in *T.brachycephala*). It can be distinguished from *T.recurva* by the shorter cephalic process curved upward about 90° in front of the eyes (the longer head curved upward about 60° in front of eyes in *T.recurva*) and the ventral lobes of the aedeagus weakly trilobed (nearly cross-shaped in *T.recurva*).

##### Description.

***Measurements*** (in mm; 1♂). Body length from apex of head to tip of tegmina: 11.7; head length (includes: apex of cephalic process to constricted and curved part + from curved part to base of eyes): 0.3+1.1; head width including eyes: 1.6; tegmen length: 9.7.

***Coloration*.** Head stramineous green, lateral carinae of vertex and frons reddish brown, intermediate carinae of frons in front of eyes black to reddish brown, lateral areas in front of eyes green. Pronotum and mesonotum stramineous green, upper lateral carinae of pronotum green. Tegmina and hindwings with membrane hyaline, costal margin dark brown, veins green to greenish yellow, pterostigmal area greenish ochraceous. Legs yellowish brown, base, apex and apical spines of tibiae fuscous. Abdomen dorsally and ventrally greenish ochraceous.

***Structure*.** Head with cephalic process short, in lateral view (Fig. 6B), curved upward nearly 90° in front of eyes. Vertex (Fig. 6A) broad, with ratio of length at midline to width between eyes (1.1–1.2):1. Frons (Fig. 6C) flat, relatively broad, with ratio of length at midline to maximum width 2.2:1, intermediate and median carinae obscure.

***Male genitalia*.** Pygofer in lateral view (Fig. 6E) with ratio of ventral to dorsal width about 2.0:1; posterior margin produced angulately near middle. Gonostyles (Fig. 6F) large and broad, dorsal process elongate, triangular, acute apically, more or less incurved and directed dorsoanteriad; hook-like process placed sub-basally, below dorsal process, curved apicad (Fig. 6G). Aedeagus (Fig. 6H–J) large and stout, endosomal processes curved dorsoanteriad; phallobase sclerotized and pigmented at lateral sides, membranous and slightly inflated dorsally and ventrally: dorsal lobes small, V-shaped at apex, directed posteriad; a pair of lateral lobes large and elongate, thumb-like, tapering apicad, apex with a large long spine, directed posterolaterad; ventral lobes large, weakly trilobed, base protruded laterad, with a large long spine, apex produced in a long triangular lobe, without spine, directed posteriad. Segment X (Fig. 6D, E), in dorsal view, with lateral margins more or less convex near middle, with ratio of length to width near middle about 2.1:1.

**Female.** Unknown.

##### Etymology.

The new species is named for its occurrence in Peru. The specific epithet ‘peruensis’ is to be treated as a Latinized adjective in nominative singular.

##### Distribution.

Southeastern Peru.

#### ﻿ Trigava recurva

Taxon classificationAnimaliaHemipteraDictyopharidae

(Melichar, 1912)

9D068499-B43E-558A-8587-8FEE5F1C3C48

Figs 1F , 2F, H , 7A–J

Igava recurva Melichar, 1912: 49, pl. II, figs 8, 10.
Igava recurva Melichar: Metcalf (1946): 39.
Trigava recurva (Melichar): O’Brien (1999): 60.

##### Type locality.

Bolivia, La Paz Department, Mapiri.

##### Emended description.

***Measurements*** (in mm; 1♂, 1♀). Body length from apex of head to tip of tegmina: ♂ 13.8, ♀ 14.1; head length (includes: apex of cephalic process to constricted and curved part + from curved part to base of eyes): ♂♀ 0.6+(0.9–1.0); head width including eyes: ♂ 1.7, ♀ 1.6; tegmen length: ♂ 11.4, ♀ 11.7.

***Coloration*.** Head stramineous green, lateral carinae of vertex reddish brown, lateral and intermediate carinae of frons in front of eyes black to reddish brown, lateral areas in front of eyes green. Pronotum and mesonotum stramineous green, upper lateral carinae of pronotum green. Tegmina and hindwings with membrane hyaline, costal margin black to dark brown, veins green to greenish yellow, pterostigmal area more or less greenish ochraceous. Legs yellowish brown, base, apex and apical spines of tibiae fuscous. Abdomen dorsally and ventrally greenish ochraceous.

***Structure*.** Head with cephalic process relatively long, in lateral view (Fig. 7B), curved upward about 60° in front of eyes. Vertex (Fig. 7A) broad, with ratio of length at midline to width between eyes (1.6–1.7):1. Frons (Fig. 7C) flat, relatively broad, with ratio of length at midline to maximum width 2.6:1.

***Male genitalia*.** Pygofer in lateral view (Fig. 7E) with ratio of ventral to dorsal width about 2.1:1; posterior margin broadly angular near middle. Gonostyles (Fig. 7F) large and broad, dorsal process elongate, triangular, acute apically, more or less incurved and directed dorsoanteriad; hook-like process situated sub-basally, slightly below dorsal process, curved apicad (Fig. 7G). Aedeagus (Fig. 7H–J) large and stout, endosomal processes curved dorsoanteriad; phallobase sclerotized and pigmented at lateral sides, membranous and slightly inflated dorsally and ventrally: dorsal lobes small, U-shaped at apex, directed posteriad; a pair of lateral lobes large and elongate, base rounded and expanded laterad, remaining thumb-like, subapex with a large long spine, directed posteriad; ventral lobes large, nearly cross-shaped in ventral view (Fig. 7J), base protruded anteriad, middle expanded laterad, with a large and stout spine, apex produced in a large thumb-like lobe, without spine, directed posteriad. Segment X (Fig. 7D, E), in dorsal view, lateral margins more or less convex near middle, with ratio of length to width near middle about 1.8:1.

***Female genitalia*.** As in generic description.

##### Type material examined.

***Lectotype*** (here designated), ♂, (1) “Bolivia, Mapiri”; (2) “*recurva* [Melichar’s handwriting], det. Melichar”; (3) “Typus” [dark red label]; (4) “Collectio Dr. L. Melichar, Moravské museum Brno”; (5) “Syntypus, *Igavarecurva* sp.n. Melichar, 1912, ♂ [P. Lauterer’s handwriting], P. Lauterer det 1991”; (6) “Syn- typus” [red label]; (7) “Invent. č. 4942/Ent., Mor. muzeum, Brno”; (8) “*Trigavarecurva* (Melichar) [Zhi-Shun Song’s handwriting] det. Z.S. Song 2014”; (9) “Lectotypus ♂, *Igavarecurva* Melichar, 1912, desig. by Z. S. Song & I. Malenovský, 2023” [newly added red label] (MMBC). ***Paralectotype***, 1♀, (1) “Bolivia N, Yungas, Garlepp c.”; (2) “Coll. A. Jacobi, 1912 – 3” [green label]; (3) “*Enhydriarecurva M*” [handwriting]; (4) “Paralectotypus ♀, *Igavarecurva* Melichar, 1912, labelled by I. Malenovský, 2023” [newly added red label] (MTD).

##### Distribution.

Northwestern Bolivia, southern Peru (Melichar 1912).

##### Remarks.

*Igavarecurva* Melichar was described based on an unspecified number of specimens from “Peru, Pachitea, Marcapata; Bolivien, Mapiri, Yungas (Garlepp) (Mus. Budapest und Dresden)” (Melichar 1912). One male syntype from Bolivia, Mapiri preserved in Melichar’s personal collection in MMBC is here used for the redescription of the species and designated as the lectotype according to Article 74 of ICZN (1999) to stabilize the nomenclature. Another conspecific female from Bolivia with collecting data matching the original description has been located by us in MTD and was labelled as a paralectotype. Specimens from Peru have not been examined by us.

## ﻿Discussion

Nersiini Emeljanov, 1983 is the second largest tribe in Dictyopharidae, comprising 26 genera from the New World, mostly distributed in the Neotropical region with a few species in the Nearctic region (Emeljanov 1983, 2011; Bartlett et al. 2014; Bartlett 2023; Bourgoin 2023). Nersiini displays the greatest disparity within Dictyopharidae, such as the massive size, carinate tegulae, relatively rich venation, piercing-cutting ovipositor, and bifurcated endosomal processes (Song et al. 2018). As in many other groups of Auchenorrhyncha, the New World dictyopharid fauna currently remains inadequately studied and most genera within Nersiini lack standard revisionary studies.

According to Song et al. (2018), Nersiini is polyphyletic. The genera *Dictyopharoidess.s.* Fowler, 1900, *Paramisia* Melichar, 1912, *Pharodictyon* Fennah, 1958, *Sicoris* Stål, 1862, and *Xenochasma* Emeljanov, 2011 should be excluded from Nersiini based on their morphology. Nersiini*s.s.* includes three clades, the *Digitocrista*^+^ clade, the *Nersia*^+^ clade and the *Trigava*^+^ clade; the last one comprises six genera: *Trigava*, *Paralappida* Melichar, 1912, *Crocodictya* Emeljanov, 2008, *Mitrops* Fennah, 1944, *Rhynchomitra* Fennah, 1944 and a new undescribed genus represented by *Dictyopharoidesinficita* Melichar, 1912 (Song et al. 2018).

In the *Trigava*^+^ clade, *Trigava* is closely related to *Paralappida*, represented by two species from Brazil, but can be easily distinguished from the latter by the following characters: the head strongly curved upward (slightly curved upward in *Paralappida*); the intermediate carinae of the frons approaching to the frontoclypeal suture (to middle of eyes in *Paralappida*); the posterior margin of the pronotum not notched (with a deep narrow notch in *Paralappida*); and the tegulae lacking a carina (present in *Paralappida*).

The four species of *Trigava* are very similar in external morphology and can be divided into two distinct lineages based on the differences in the male genitalia. Within the *brachycephala* lineage including *T.brachycephala* and *T.obrieni* sp. nov., the gonostyles have a shorter dorsal process and the hook-like process situated submedially and curved basad, and the ventral lobes of the aedeagus lack long spines; while in the *recurva* lineage including *T.recurva* and *T.peruensis* sp. nov., the gonostyles have an elongate dorsal process and the hook-like process situated more basally and curved apicad, and the ventral lobes of the aedeagus possess a pair of long spines.

## Supplementary Material

XML Treatment for Trigava

XML Treatment for Trigava brachycephala

XML Treatment for Trigava obrieni

XML Treatment for Trigava peruensis

XML Treatment for Trigava recurva

## ﻿Additional information

### Author ORCIDs

Zhi-Shun Song https://orcid.org/0000-0002-5449-4646

Igor Malenovský https://orcid.org/0000-0001-8840-2263

Jürgen Deckert https://orcid.org/0000-0003-4211-4463

Charles R. Bartlett https://orcid.org/0000-0001-9428-7337

### Data availability

All of the data that support the findings of this study are available in the main text.
